# Successful Treatment of an Extensive Deep Vein Thrombosis in the Lower Left Extremity Through Thrombectomy and Endovascular Stenting: Role of May-Thurner Syndrome in Differential Diagnoses

**DOI:** 10.7759/cureus.82468

**Published:** 2025-04-17

**Authors:** Hong Thoai Nguyen, Chau Dang, Juveriya Yasmeen, Pham Thao Vy Le, Lac Han Nguyen

**Affiliations:** 1 Internal Medicine, Ascension Saint Joseph Hospital, Chicago, USA; 2 Internal Medicine, San Joaquin General Hospital, French Camp, USA; 3 Internal Medicine, University of Texas at Tyler, Tyler, USA

**Keywords:** deep vein thrombosis (dvt), endovascular stenting, endovascular thrombectomy, iliac compression syndrome, may-thurner syndrome

## Abstract

May-Thurner syndrome (MTS) is a vascular condition caused by extrinsic compression of the left common iliac vein by the right common iliac artery, predisposing patients to deep vein thrombosis (DVT). Though often asymptomatic, MTS can present with unilateral leg swelling and pain, particularly in young women without traditional risk factors. We present the case of a 45-year-old woman who developed acute left lower extremity DVT without prior medical history or provoking factors. Imaging confirmed extensive thrombosis and revealed complete iliac vein compression consistent with MTS. The patient underwent successful mechanical thrombectomy, stenting, and balloon angioplasty, followed by long-term anticoagulation. Two years post-intervention, she exhibited chronic venous changes but no recurrent DVT. This case highlights the importance of early diagnosis and endovascular management in symptomatic MTS to optimize outcomes and reduce long-term complications.

## Introduction

May-Thurner syndrome (MTS) arises from extrinsic compression of the iliac vein, most commonly involving the left common iliac vein as it is compressed between the overlying right common iliac artery and the underlying lumbar spine [1]. While this anatomical variant is often clinically silent, symptomatic cases can manifest with lower extremity discomfort, extensive deep vein thrombosis (DVT), and cutaneous changes associated with post-thrombotic syndrome. Management strategies are guided by the severity of clinical presentation and the presence of thrombosis, incorporating systemic anticoagulation, catheter-directed thrombolysis, endovascular angioplasty, stent placement, open surgical thrombectomy, and other interventional approaches [2]. In the case presented in this article, early identification and timely intervention facilitated optimal therapeutic outcomes, minimizing the risk of treatment resistance and recurrent thrombotic complications.

## Case presentation

**Figure 1:**
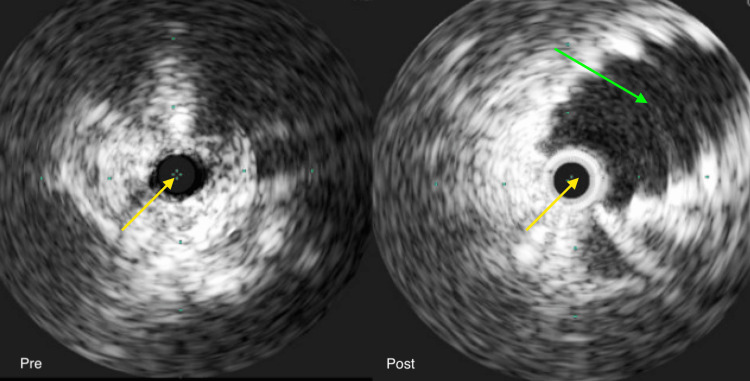
Intravenous ultrasound showed a collapsed left iliac vein pre-intervention and an expanded left iliac vein (green arrow) post-intervention. The yellow arrows are pointing out the ultrasound probe. Green dots are measurement markers.

A 45-year-old woman with no significant past medical history presented with a three-day history of pain and swelling in the left lower extremity. The patient denied associated symptoms, including dyspnea, cough, chest pain, fever, abdominal pain, nausea, vomiting, diarrhea, or dysuria. She had no personal or family history of DVT and no history of prolonged immobilization. Venous duplex ultrasonography revealed extensive thrombus burden with the absence of flow within the proximal, mid, and distal segments of the femoral vein and popliteal vein and portions of the posterior tibial and peroneal veins. Computed tomography (CT) angiography demonstrated thrombus extension into the left common femoral, superficial femoral, popliteal, and anterior and posterior tibial veins, with no evidence of propagation into the iliac venous system or inferior vena cava. A hypercoagulability workup was unremarkable (Table 1). Management included anticoagulation with enoxaparin and endovascular intervention via mechanical thrombectomy utilizing the Inari ClotTriever system. Intravascular ultrasound (IVUS) identified MTS, with complete compression (0 mm luminal diameter) of the left common iliac vein at the crossing of the right common iliac artery. Consequently, an 18 mm × 100 mm Venovo self-expanding stent was deployed from the caval confluence to the proximal external iliac vein, followed by post-dilation with a 16 mm × 40 mm balloon angioplasty (Figure 1). Post-procedural venography confirmed full restoration of venous flow without residual stenosis. The patient was prescribed indefinite anticoagulation with apixaban. Two years after the initial presentation, the patient continued to exhibit trace pedal edema of the left lower extremity. Repeat venous duplex ultrasonography revealed the presence of collateral circulation, with absent flow in the proximal and mid-segments of the left femoral vein, findings suggestive of chronic venous occlusion secondary to prior thrombotic events; no evidence of acute DVT was observed in the left lower extremity.

**Table 1:** Hypercoagulability workup

Component	Reference range	Result
Anticardiolipin IgA ([APL'U]/mL)	0.0 - 19.9	1.0
Anticardiolipin IgG ([GPL'U]/mL)	0.0 - 19.9	<1.6
Anticardiolipin IgM ([MPL'U]/mL)	0.0 - 19.9	1.0
Beta-2-Glycoprotein IgG (U/mL)	0.0 - 19.9	<1.4
Beta-2-Glycoprotein IgM (U/mL)	0.0 - 19.9	1.0

## Discussion

MTS, also referred to as ilio-caval compression syndrome or iliac vein compression syndrome, is a vascular condition characterized by the extrinsic compression of the ilio-caval venous structure by the overlying arterial system against the adjacent bony framework [1]. Among its various anatomical subtypes, the most frequently encountered variant involves the compression of the left common iliac vein by the right common iliac artery. Although MTS can remain clinically silent, symptomatic presentations typically manifest as ipsilateral lower extremity pain, edema, and cutaneous alterations. The syndrome may present in either a thrombotic or nonthrombotic form; however, it should be included in the differential diagnosis of individuals presenting with iliofemoral DVT [2].

Rudolf Virchow initially described the compression of the left iliofemoral vein by the right common iliac artery in 1851 based on cadaveric examinations of individuals with left iliofemoral thrombosis. However, it was not until 1957 that May and Thurner identified the presence of intraluminal fibrous bands within the left iliofemoral vein, which were compressed by the overlying right common iliac artery in 22% of 430 cadaveric dissections; these structures were subsequently termed "spurs," and the associated condition became known as MTS [2]. The first documented clinical series of 57 patients presenting with acute left iliofemoral DVT secondary to iliac vein compression was reported by Cockett et al. in 1965 and 1967 [3,4]. In recent years, the recognition of this vascular anomaly has increased due to heightened physician awareness, a growing body of research, and advancements in imaging modalities. Additionally, the role of endovascular interventions in the management of this condition has expanded considerably [5].

Chronic mechanical compression of the iliac vein can trigger an inflammatory response within the vessel wall, leading to the accumulation of elastin and collagen types I and III, as well as smooth muscle cell proliferation. This cascade of events results in intimal fibrosis, which may progressively narrow the lumen and disrupt normal venous flow, contributing to the development of unilateral venous hypertension and an increased risk of venous thrombosis [6].

Many individuals with MTS remain asymptomatic due to the presence of venous collaterals that maintain adequate venous drainage. However, DVT is most commonly observed in patients with MTS when additional risk factors such as venous stasis, vessel injury, or a prothrombotic condition are present [1].

The clinical presentation of MTS varies significantly among patients. Some may develop a gradual progression of venous insufficiency, while others experience an acute onset of lower extremity pain and swelling. The most commonly reported presentation involves younger women who present with left lower extremity swelling, which improves with rest and elevation. Over time, this can progress to dermal hyperpigmentation and ulceration. In other cases, symptoms may appear after surgery, during or shortly after pregnancy, or upon starting oral contraceptives, with the primary symptom being painful, swollen left lower extremities. Rarely, both men and women may experience more severe complications, such as phlegmasia cerulea dolens or other limb-threatening conditions [7].

The patient presented with an acute onset of marked swelling and pain localized to the left lower extremity. Notably, she lacked any significant past medical history or identifiable provoking factors, which is consistent with the typical clinical profile observed in MTS. This diagnosis is further supported by the higher prevalence of MTS in women. As this represents her initial presentation, there is no clinical evidence of chronic venous insufficiency sequelae, such as dermal hyperpigmentation or venous ulceration, which are commonly associated with post-thrombotic syndrome in more advanced or recurrent cases.

Doppler ultrasonography is typically the first imaging method used to assess venous insufficiency or distal thrombus. If ultrasonography does not provide clear results for the proximal vessels due to factors like body habitus or obstructing structures, CT or magnetic resonance (MR) venography is recommended [8]. CT venography is highly effective, with over 95% sensitivity and specificity for detecting iliac vein compression. It can also show stenosis, collateral circulation, and DVT. Additionally, CT venography can help rule out other causes of iliac vein compression, such as lymphadenopathy, malignancy, or hematoma [9]. For patients who cannot undergo CT, such as those with pregnancy or renal issues, MR venography is a good alternative, offering better visualization of structures. However, MR venography may not always detect MTS, as compression of the common iliac vein can be intermittent and variable. MR venography is also more expensive and less readily available than Doppler ultrasonography or CT venography [10]. Intravascular ultrasonography has mostly replaced catheter venography for cases where noninvasive methods are inconclusive. Unlike catheter venography, which carries risks like contrast use, radiation, and bleeding, intravascular ultrasonography has over 98% sensitivity for iliac vein compression and provides detailed information on stenosis, fibrosis, and thrombus [11]. It is also useful for precise measurements, stent preparation, guidewire visualization during recanalization, and confirming stent placement. In addition, intravascular ultrasonography is the only technique that can detect and quantify subtle vessel changes [12].

Initial diagnostic evaluation with venous duplex ultrasonography of the left lower extremity revealed an extensive thrombotic burden, characterized by absent venous flow throughout the proximal, mid, and distal segments of the femoral vein, as well as involvement of the popliteal vein and portions of the posterior tibial and peroneal venous systems. Given the significant thrombus load and the need for precise delineation of the proximal extent of involvement, CT venography was subsequently performed to further characterize the thrombosis and to guide consideration for potential thrombectomy.

The treatment of MTS depends on the clinical presentation and the presence of a thrombus. In cases without thrombus, angioplasty and venous stenting are typically recommended. For patients with mild symptoms, conservative management, such as compression, limb elevation, counseling on prothrombotic risks, and regular follow-up, is generally sufficient. When a thrombus is present and the patient has an acceptable risk for anticoagulation, anticoagulation or antiplatelet therapy is advised [13]. The standard anticoagulation approach starts with unfractionated heparin, followed by a switch to low-molecular-weight heparin or fondaparinux as a bridge to warfarin or novel oral anticoagulants [14]. Anticoagulation alone, however, is not adequate for managing iliofemoral vein thrombosis caused by MTS. Studies have shown that vascular angioplasty without stenting is often insufficient due to irreversible venous stenosis in these patients [15,16]. For those who cannot undergo thrombolytic therapy, open thrombectomy followed by angioplasty and stenting is a preferred option [17]. Surgical resection of the affected vein is rare and reserved for patients who do not respond to endovascular treatments. Surgical procedures available include bypass surgeries and aortic elevation [18].

The patient was initially managed with subcutaneous enoxaparin and subsequently underwent mechanical thrombectomy for endovascular intervention. IVUS revealed a diagnosis of MTS, demonstrating complete luminal collapse (0 mm diameter) of the left common iliac vein at the site of compression beneath the overlying right common iliac artery, an abnormality not visualized on prior CT venography. In response, an 18 mm × 100 mm Venovo self-expanding stent was placed extending from the inferior vena cava confluence to the proximal segment of the external iliac vein. This was followed by post-stent balloon angioplasty utilizing a 16 mm × 40 mm balloon. Completion venography confirmed the restoration of unobstructed venous flow with no evidence of residual stenosis. The patient was transitioned to indefinite anticoagulation with apixaban.

MTS is often asymptomatic, but when symptoms occur, early diagnosis and intervention to relieve venous compression are essential for maintaining a good quality of life. Regular follow-up is necessary to monitor symptoms and detect complications early. Close clinical surveillance can help reduce the risk of recurrent thrombosis and improve long-term outcomes [1]. Post-thrombotic syndrome is a common complication in symptomatic patients, but its incidence can be minimized to less than 10% with appropriate treatment [19]. Mechanical or pharmacologic thrombolysis may lower this risk, and additional measures such as wearing knee- or thigh-high compression stockings and following a structured exercise program can further support symptom management [20].

The patient continues to engage in routine follow-ups with her medical providers. Although her edema has improved, she continues to exhibit trace swelling accompanied by mild discomfort. A repeat duplex ultrasonography of the left lower extremity revealed evidence of collateral venous formation, with absent flow in the proximal and mid-segments of the femoral vein, findings suggestive of chronic thrombotic occlusion. No evidence of acute DVT was identified. Given these findings, the patient remains at risk for developing post-thrombotic syndrome and will require ongoing surveillance to mitigate this potential complication. MTS should remain a key consideration in the differential diagnosis of patients presenting with unilateral lower extremity deep vein thrombosis, particularly in the absence of typical risk factors. Timely recognition and appropriate intervention are essential, as delayed diagnosis may contribute to suboptimal responses to anticoagulation therapy. Early endovascular or surgical management has been associated with improved clinical outcomes and a reduced risk of recurrence [17].

## Conclusions

MTS is a vascular condition in which the left common iliac vein is compressed by the right common iliac artery, typically at the level of the lumbar spine. The most common presentation involves compression of the left iliac vein by the right iliac artery. Many patients remain asymptomatic for extended periods, but some may eventually develop significant DVT. Ultrasound is often the first diagnostic tool, followed by other non-invasive imaging techniques to confirm the diagnosis. When initial imaging is unclear or when preparing for stent placement, intravenous ultrasound may be employed for better visualization. The mainstay of treatment involves long-term anticoagulation therapy along with stenting, which has been shown to be effective in managing the condition. With improved awareness among clinicians and advances in imaging, MTS is now more frequently diagnosed at an earlier stage, allowing for interventions like intravascular stenting to reduce the risks of anticoagulation failure, recurrence, and other complications.
